# Supplementary material for: Elevated Levels of Circulating Hsp70 and an Increased Prevalence of CD94+/CD69+ NK Cells Is Predictive for Advanced Stage Non-Small Cell Lung Cancer
Source: Cancers (Basel). 2022 Nov 21;14(22):5701. doi: 10.3390/cancers14225701 (PMC9688749; doi:10.3390/cancers14225701)
Supplement: Supplementary file 1 [file cancers-14-05701-s001.zip › cancers-1989692-supplementary.pdf]

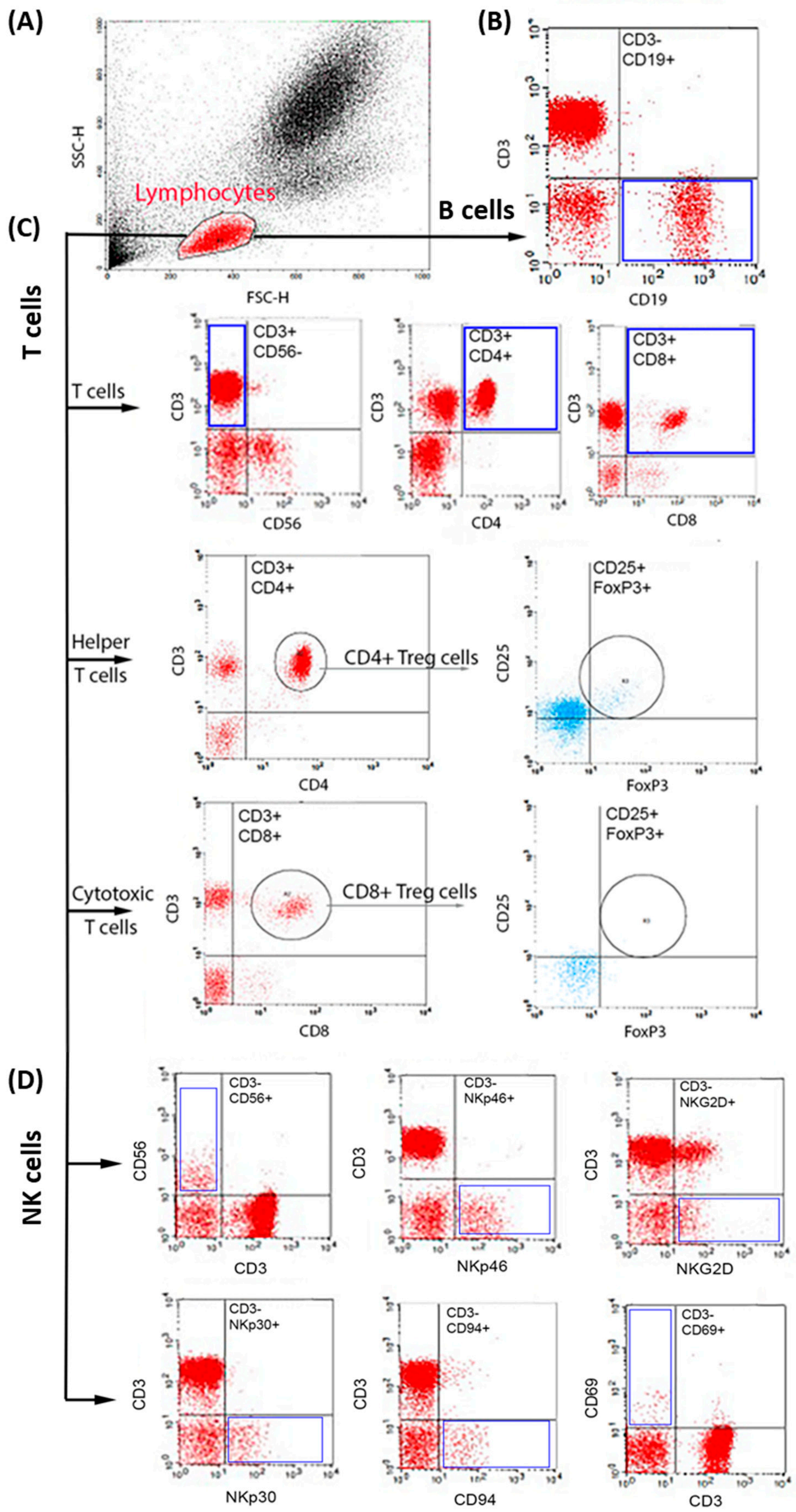

Figure S1. Representative gating strategy for identifying lymphocyte subpopulations and their expressions. The gating strategy involved the identification of lymphocyte based on **(A)** FSC vs. SSC, followed by separation of the **(B)** CD3−/CD19+ B cell subpopu-

lation, **(C)** CD3+ T cell subpopulations, and **(D)** CD3- NK cell subpopulations. Flow cytometry plots show receptor expression relative to their counterparts incubated with the respective isotype-matched control mAb.
